# MicroRNA Signature in an In Vitro Keratinocyte Model of Diabetic Wound Healing

**DOI:** 10.3390/ijms251810125

**Published:** 2024-09-20

**Authors:** Hsin-Chung Tsai, Gary Ro-Lin Chang, Min-Che Tung, Min-Yu Tu, I-Chien Chen, Yu-Hsien Liu, Abdulkadir Cidem, Chuan-Mu Chen

**Affiliations:** 1 Department of Life Sciences, Doctorial Program in Translational Medicine, National Chung Hsing University, Taichung 402, Taiwan; prch1212@gmail.com (H.-C.T.); gary590422@yahoo.com.tw (G.R.-L.C.); du0807@yahoo.com.tw (M.-Y.T.); chien@leon-bio.com.tw (I.-C.C.); yuhsien000@yahoo.com.tw (Y.-H.L.); cidema.kadir@gmail.com (A.C.); 2 Department of Surgery, Taichung Hospital, Ministry of Health and Welfare, Taichung 403, Taiwan; 3 Department of Surgery, Tungs’ Taichung Metro Harbor Hospital, Taichung 435, Taiwan; tungminche@gmail.com; 4 Department of Orthopedic Surgery, Taichung Armed Forces General Hospital, Taichung 411, Taiwan; 5 Department of Internal Medicine, Jen-Ai Hospital, Dali Branch, Taichung 402, Taiwan; 6 Department of Molecular Biology and Genetics, Erzurum Technical University, Erzurum 25250, Turkey; 7 The iEGG and Animal Biotechnology Research Center, National Chung Hsing University, Taichung 402, Taiwan; 8 Rong Hsing Research Center for Translational Medicine, Taichung Veterans General Hospital, Taichung 407, Taiwan; 9 Center for General Educational, National Quemoy University, Kinmen 892, Taiwan

**Keywords:** microRNAs (miRNAs), diabetic wound healing, keratinocytes, miR-3138 mimic, miR-3679-5p inhibitor, gene ontology (GO)

## Abstract

Treating diabetic wounds effectively remains a significant clinical challenge. Emerging studies suggest that microRNAs (miRNAs) play crucial roles in various physiological and pathological processes and hold promise as therapeutic tools. This study investigates the miRNA expression profile in keratinocytes using a cell model of diabetic wounds. Microarray analysis revealed that 43 miRNAs from wounded keratinocytes incubated under diabetic conditions (high glucose/hypoxia) exhibited a two-fold change in expression compared to those incubated under normal conditions (low glucose/normoxia). Quantitative RT-PCR confirmed significant differences in the expression of eight miRNAs, with miR-3138 and miR-3679-5p being further analyzed for their roles in keratinocyte migration. Transfection with a miR-3138 mimic and a miR-3679-5p inhibitor indicated that upregulation of miR-3138 and downregulation of miR-3679-5p enhance keratinocyte migration in both normal and diabetic wounds. Pathway and gene ontology (GO) analyses identified potential pathways and functional annotations associated with miR-3138 and miR-3679-5p in diabetic wound healing. Potential human gene targets of miR-3138 and miR-3679-5p were predicted using a three-way comparison of the TargetScan, miRDB, and DIANA databases. This study elucidates the miRNA expression signature of human keratinocytes in a diabetes-like environment, providing deeper insights into the pathogenesis of diabetic wounds.

## 1. Introduction

Diabetes mellitus (DM) is a complex metabolic disorder characterized by chronic hyperglycemia, leading to severe long-term complications that significantly impact patients’ quality of life and healthcare economics. One of the most prevalent and challenging complications in DM patients is impaired wound healing, which manifests as reduced keratinocyte and fibroblast migration and proliferation, limited angiogenesis, and decreased recruitment of bone marrow-derived endothelial progenitor cells [1,2]. These impairments can result in the development of chronic diabetic wounds, such as diabetic foot ulcers (DFU), which often necessitate amputation [3].

The pathogenesis of chronic diabetic wounds is multifactorial, involving a hyperglycemic environment, hypoxia, chronic inflammation, micro- and macro-circulatory dysfunction, autonomic and sensory neuropathy, and impaired neuropeptide signaling [4]. Studies have demonstrated that a high-glucose environment disrupts normal keratinocyte functions [5,6] and leads to poor angiogenesis and prolonged inflammation in diabetic wounds [7]. Despite significant advancements in modern medicine, the effective treatment of diabetic wounds remains a substantial clinical challenge.

MicroRNAs (miRNAs) have emerged as crucial regulators in various physiological and pathological processes, including wound healing. These endogenous, approximately 22-nucleotide non-coding RNAs suppress the expression of protein-coding genes by targeting mRNAs for degradation or translational repression [8]. Since their discovery in 1993, over 2000 human miRNAs have been registered in the miRBase database [9,10]. While the expression and function of many miRNAs remain to be fully elucidated, they have been shown to play essential roles in development and physiological homeostasis [11].

Normal wound healing progresses through four distinct but overlapping stages: hemostasis, inflammation, proliferation, and remodeling [12]. The migration and proliferation of keratinocytes from wound edges are crucial for wound re-epithelialization during the proliferation stage. While the molecular mechanisms leading to keratinocyte dysfunction in diabetic wounds have been extensively studied [6,7,13], the miRNA network in keratinocytes underlying diabetic wound healing remains to be further elucidated. There are growing numbers of miRNAs that have been proven to play an important role in normal wound healing and the pathogenesis of non-healing diabetic wounds [14,15,16]. For instance, miR-21, the most frequently upregulated miRNA in human cancers [17], has been found to promote keratinocyte migration and re-epithelialization in diabetic wounds by targeting PTEN and activating the AKT/ERK signaling pathway [18]. Another study demonstrated that miR-26a was upregulated in wounds of diabetic mice, and treatment with a miR-26a inhibitor could effectively enhance wound healing by activating BMP/SMAD1/ID1 signaling pathways, thus restoring endothelial cell growth and angiogenic function [19]. Furthermore, it was found that reduced expression of miR-146a is closely related to chronic inflammation in wounds of diabetic mice, and treatment with mesenchymal stem cells can enhance wound closure by increasing miR-146a levels and reducing inflammatory responses [20]. 

In this study, we aimed to elucidate the miRNA signature in diabetic wound healing using human keratinocytes (HaCaT) cultured under high-glucose and hypoxic conditions to simulate the environmental factors surrounding diabetic wounds. We employed scratch assays and miRNA microarray analysis to study the miRNA expression profile, complemented by cDNA microarray and bioinformatic tools to clarify miRNA-affected genes and predict the targets of specific miRNAs. This comprehensive approach allows for a deeper understanding of the complex regulatory networks involved in diabetic wound healing and may pave the way for novel therapeutic strategies.

## 2. Results

### 2.1. In Vitro Wound Healing and miRNA Microarray Analysis

**Figure 1:**
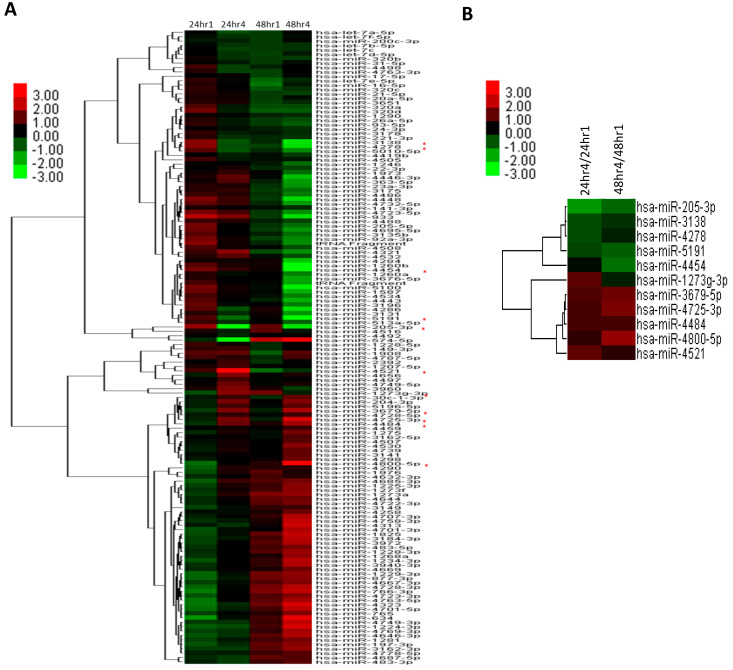
Differential expression of miRNAs in HaCaT cells under high-glucose/hypoxia versus normal conditions. (**A**) Heat map and cluster dendrogram of 143 differentially expressed miRNAs. Columns represent miRNA expression at 24 h and 48 h under normal (24 hr1 and 48 hr1) and high-glucose/hypoxia (24 hr4 and 48 hr4) conditions. (**B**) Heat map and cluster dendrogram of 11 miRNAs with the highest changes on the expression levels at 24 and 48 h post-wounding (labeled with red asterisks in (**A**)). This heat map shows miRNA changes after the normalization of the high-glucose/hypoxia condition over the normal condition. Subsequent qRT-PCR verification on these miRNAs is shown in Figure 2.

Keratinocyte migration and proliferation are critical processes in normal and diabetic wound healing. We employed an in vitro scratch wound healing assay and miRNA microarray analysis to characterize the miRNA profile associated with keratinocyte function during diabetic wound healing. Analysis of over 2500 unique human mature miRNAs revealed differential expression of 143 miRNAs. Notably, 22 miRNAs (19 upregulated and 3 downregulated) at 24 h post-wounding and 27 miRNAs (3 upregulated and 24 downregulated) at 48 h post-wounding were identified as significantly altered, with 6 miRNAs consistently differentially expressed at both time points (Table 1 and Figure 1A). Among these, the top 11 miRNAs with significant variation were selected for further verification by quantitative reverse transcription PCR (qRT-PCR) (Figure 1B).

**Figure 2:**
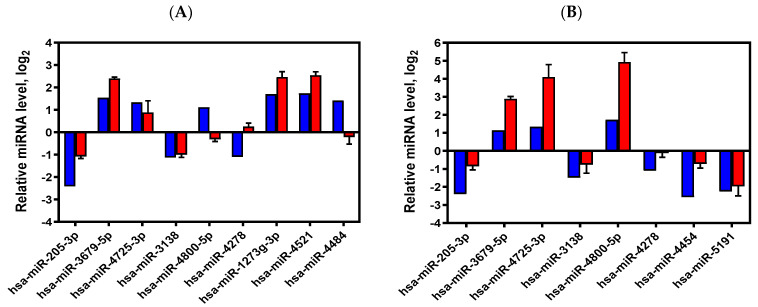
Verification of the expression patterns of the cDNA microarray by qRT-PCR. (**A**) Differentially expressed miRNAs at 24 h post-wounding. (**B**) Differentially expressed miRNAs at 48 h post-wounding. A total of 11 miRNAs were selected for further verification by qRT-PCR in this study. In the figures, the relative miRNA expression levels are all expressed on a log2 scale, with data from the cDNA microarray shown in blue, and data from three independent experiments of qRT-PCR shown in red.

After qRT-PCR verification, miR-205-3p and miR-3138 were confirmed for downregulation, and miR-3679-5p and miR-4725-3p were confirmed for upregulation at both 24 and 48 h post-wounding (Figure 2). miR-1273g-3p and miR-4521 were confirmed to be upregulated only at 24 h, and miR-4800-5p was confirmed to be upregulated only at 48 h post-wounding; meanwhile, miR-4454 and miR-5191 were confirmed for downregulation only at 48 h post-wounding. However, qRT-PCR data indicated that miR-4278 and miR-4484 expression was not significantly altered (|log2 FC| < 1), showing inconsistency with previous microarray data.

### 2.2. Modulation of miR-3138 and miR-3679-5p Enhances Keratinocyte Migration

**Figure 3:**
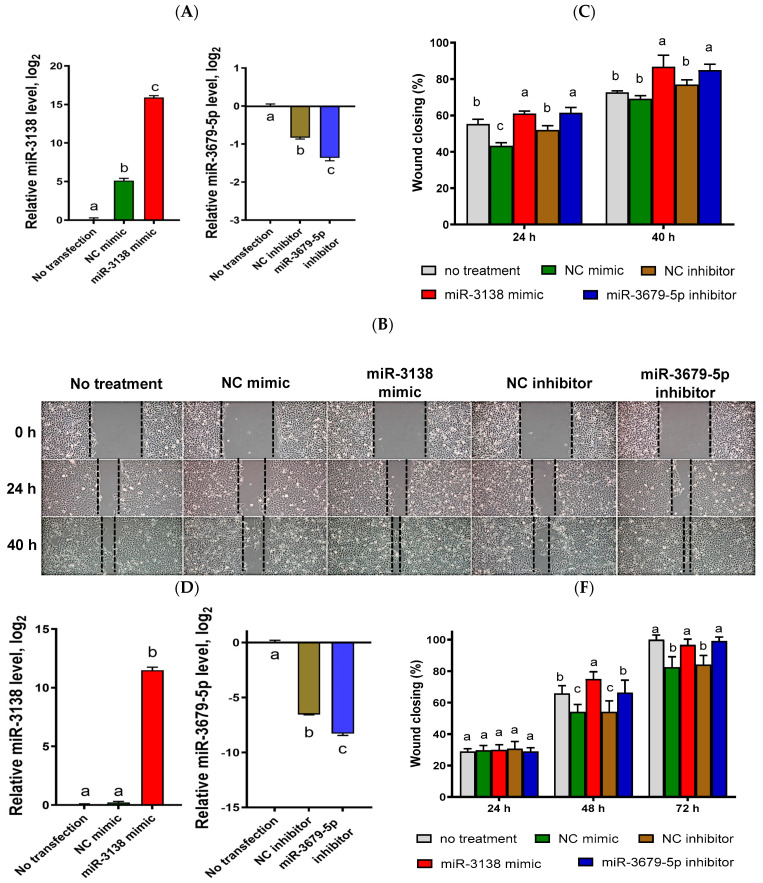

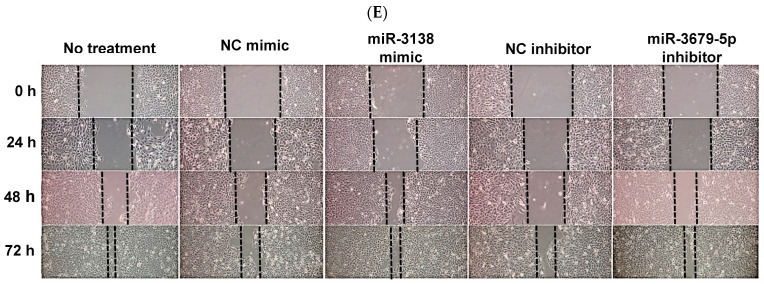
Effects of miR-3138 mimic and miR-3679-5p inhibitor transfection on keratinocyte migration. The present experiments were performed under normal (**A**–**C**) and high-glucose/hypoxic conditions (**D**–**F**). (**A**,**D**) Relative miR-3138 and miR-3679-5p levels post-transfection. (**B**,**E**) Representative images of keratinocyte migration after the transfection with miRNA mimic and inhibitor. (**C**,**F**) Average wound closure percentages from three independent experiments after the transfection with miRNA mimic and inhibitor. Different letters labeled at the top of columns indicate statistically significant differences (*p* < 0.05).

Due to persistent upregulation or downregulation at 24 and 48 h and their functions in the regulation of diabetic wound healing having been rarely studied, miR-3138 (downregulation) and miR-3679-5p (upregulation) were selected to further characterize their possible roles during diabetic wound healing. The related experiments were conducted by transfection of a miR-3138 mimic or a miR-3679-5p inhibitor into HaCaT cells to evaluate their impact on cell migration and proliferation. Successful transfection was confirmed by qRT-PCR, demonstrating upregulation of miR-3138 and downregulation of miR-3679-5p in HaCaT cells under both normal and diabetic conditions (Figure 3A,D).

Scratch wound healing assays revealed that cells transfected with the miR-3138 mimic or the miR-3679-5p inhibitor migrated faster than non-transfected cells and those transfected with negative control (NC) mimic and inhibitor (Figure 3B,E). Under normal conditions, wound closure in miR-3138 mimic-treated cells was significantly higher (61 ± 1% at 24 h and 87 ± 6% at 40 h) compared to non-transfected cells (55 ± 3% and 73 ± 1%) and NC mimic-transfected cells (43 ± 2% and 69 ± 2%). Similarly, miR-3679-5p inhibitor-transfected cells showed enhanced wound closure (61 ± 3% at 24 h and 85 ± 3% at 40 h) compared to NC inhibitor-transfected cells (52 ± 2% and 77 ± 3%) (Figure 3C).

Under diabetic conditions (high glucose and hypoxia), keratinocyte migration was generally decreased. However, miR-3138 mimic-transfected cells still exhibited significantly enhanced wound closure (30 ± 3% at 24 h, 75 ± 5% at 48 h, and 97 ± 4% at 72 h) compared to non-transfected cells (29 ± 2%, 66 ± 5%, and 100 ± 3%) and NC mimic-transfected cells (30 ± 3%, 54 ± 5%, and 83 ± 7%). Likewise, miR-3679-5p inhibitor-transfected cells showed significantly improved wound closure (29 ± 2%, 66 ± 8%, and 99 ± 3%) compared to NC inhibitor-transfected cells (31 ± 5%, 54 ± 7%, and 84 ± 6%) (Figure 3F).

These results suggest that miR-3138 and miR-3679-5p may play significant roles in the pathogenesis of diabetic wounds, and modulation of their expression may provide potential therapeutic approaches for non-healing diabetic wounds.

### 2.3. Kyoto Encyclopedia of Genes and Genomes (KEGG) Pathway and Gene Ontology (GO) Analysis

**Figure 4:**
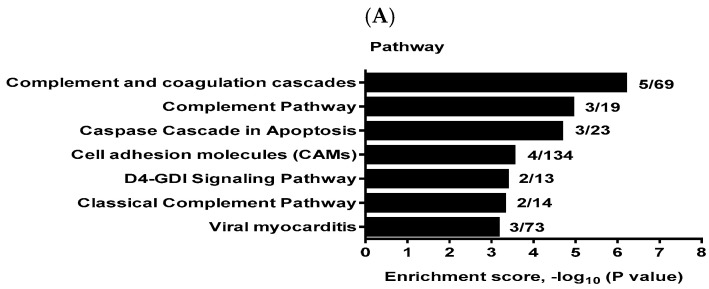

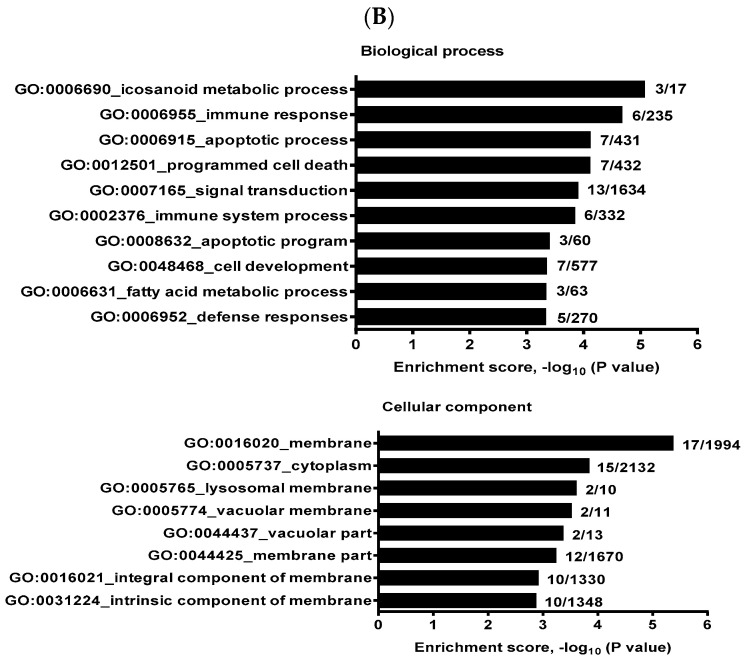
KEGG pathway and GO analysis of HaCaT cells transfected with miR-3138 mimic. (**A**) Enriched KEGG pathways. (**B**) Top 10 GO terms in the biological process and cellular components of GO analysis. Results are ranked by enrichment score, and the numbers shown on the right side of each histogram indicate the overlapped gene number and the total gene number in the gene set.

To elucidate the pathways and functions affected by miR-3138 and miR-3679-5p, we performed global cDNA microarray analysis with gene set enrichment analysis. Differentially expressed mRNAs affected by miR-3138 were enriched in KEGG pathways including complement, apoptosis, cell adhesion, D4-GDP dissociation inhibitor (GDI) signaling, and viral myocarditis (Figure 4A). 

GO analysis revealed that the top 10 biological processes affected by miR-3138 included apoptosis-related processes (GO:0006915/0012501/0008632), immune system response (GO:0006955), signal transduction (GO:0007165), cell development (GO:0048468), defense response (GO:0006952), and lipid metabolism (GO:0006690/0006631). The most affected cellular components annotated seven membrane-related GO terms (GO: 0016020/0005765/0005774/0044437/0044425/0016021/0031224) and one cytoplasm-related GO term (GO:0005737) (Figure 4B). There were none annotated in the molecular function component of GO analysis.

**Figure 5:**
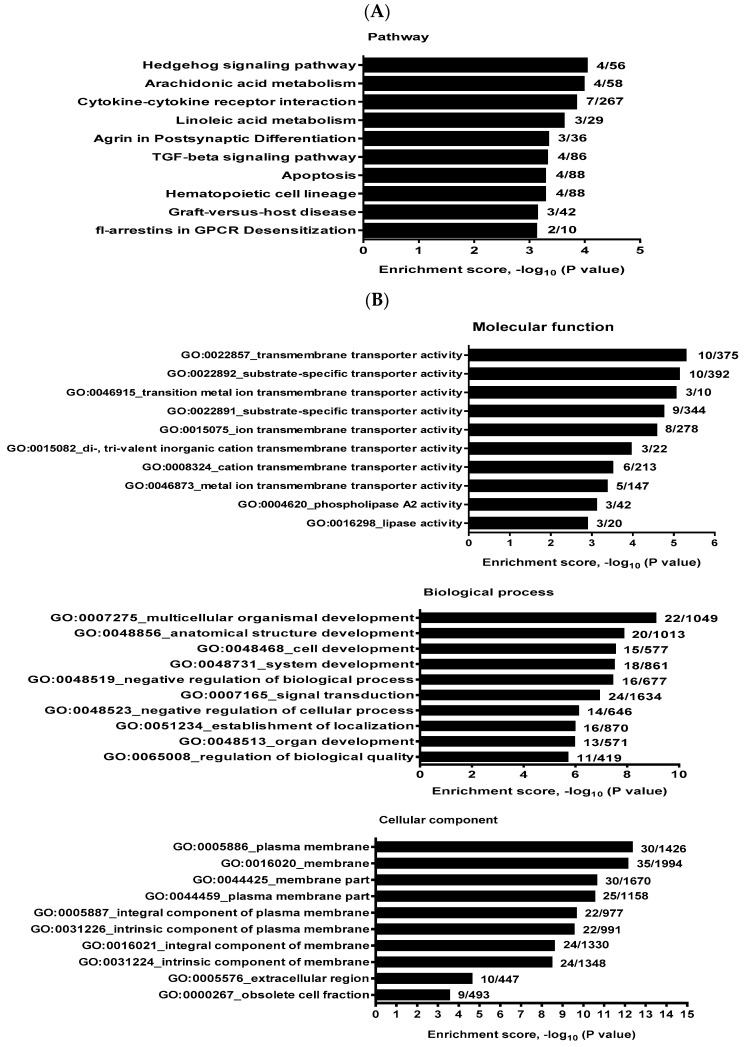
KEGG pathway and GO analysis of HaCaT cells transfected with miR-3679-5p inhibitor. (**A**) Top 10 enriched KEGG pathways. (**B**) Top 10 enriched GO terms in each component of GO analysis. Results are ranked by enrichment score, and the numbers shown on the right side of each histogram indicate the overlapped gene number and the total gene number in the gene set.

For miR-3679-5p, the top three KEGG pathways pointed to Hedgehog signaling, arachidonic acid metabolism, and cytokine–cytokine receptor interaction (Figure 5A). The top 10 enrichments in the molecular function component of GO analysis are mainly related to membrane transporter (GO:0022857/0022892/0046915/0022891/0015075/0015082/0008324/0046873) and lipase activities (GO:0004620/0016298). The biological process component annotated most of the GO terms on development (GO:0007275/0048856/0048468/0048731/0048513), next regulation of biological processes (GO:0048519/0048523) and signal transduction (GO:0007165). The cellular component of GO analysis annotated 8 of the top 10 enrichments on membrane-related terms (GO:0005886/0016020/0044425/0044459/0005887/0031226/0016021/0031224) (Figure 5B).

### 2.4. Target Gene Prediction

**Figure 6:**
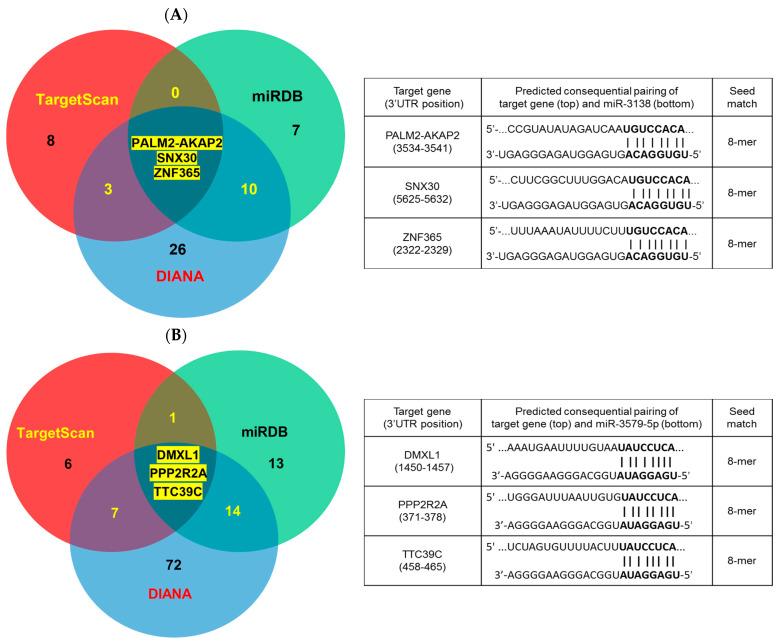
Bioinformatic prediction of the target genes for miR-3138 (**A**) and miR-3679-5p (**B**). Venn diagrams show overlapped target numbers predicted by TargetScan (red), miRDB (green), and DIANA (blue) databases. The pairing patterns between the sequences of target gene 3’UTR and miRNA are shown below each diagram.

Using TargetScan, miRDB, and DIANA databases, we predicted potential target genes for miR-3138 and miR-3679-5p. miR-3138 was predicted to target PALM2-AKAP2 (PALM2-AKAP2 readthrough), SNX30 (sorting nexin family member 30), and ZNF365 (zinc finger protein 365) (Figure 6A). miR-3679-5p was predicted to target DMXL1 (Dmx-like 1), PPP2R2A (protein phosphatase 2, regulatory subunit B, α isoform), and TTC39C (tetratricopeptide repeat domain 39C) (Figure 6B). All predicted interactions were based on complementary octamer sequences between the 5‘ region of the miRNA and the 3‘UTR of the target gene.

## 3. Discussion

In this study, we explored the miRNA signature of keratinocytes in a diabetic wound model. Comparison between high-glucose/hypoxia and normal conditions revealed that 22 miRNAs were differentially expressed at 24 h post-wounding and 27 miRNAs at 48 h post-wounding (Table 1). Verification by qRT-PCR confirmed the downregulation of miR-3138 and the upregulation of miR-3679-5p in this diabetic wound model. Functional characterization through the delivery of miRNA mimics and inhibitors demonstrated that miR-3138 mimic and miR-3679-5p inhibitor significantly enhanced keratinocyte migration under both normal and high-glucose/hypoxia conditions. These findings suggest that miR-3138 and miR-3679-5p may play critical roles in keratinocyte function during diabetic wound healing (Figure 3).

Using cDNA microarray and gene set enrichment analysis, we identified pathways and GO terms affected by miR-3138 and miR-3679-5p. miR-3138 was predicted to target genes such as PALM2-AKAP2 readthrough, sorting nexin family member 30, and zinc finger protein 365, while miR-3679-5p was predicted to target Dmx-like 1, protein phosphatase 2, and tetratricopeptide repeat domain 39C (Figure 6). These targets were identified through matching cDNA expression patterns with TargetScan, miRDB, and DIANA databases.

Previous studies have also identified miRNA signatures of diabetic wound healing in different animal models and clinical patients. Madhyastha et al. [21] identified an altered miRNA signature in diabetic mouse wounds and demonstrated the role of miR-21 in fibroblast migration during wound healing. Liu et al. [22] revealed differentially expressed miRNAs in streptozotocin (STZ)-induced diabetic rats, implicating MAPK, Wnt, and other signaling pathways in wound healing. Bhattacharya et al. [23] reported a downregulated miRNA signature in STZ-induced diabetic rat wounds during the prolonged inflammatory phase, linking miRNA downregulation to decreased Dicer levels and specific wound-healing gene networks. Dangwal et al. [24] explored circulating miRNA signatures in type 2 diabetic patients with peripheral arterial disease and chronic wounds, indicating that inflammation in chronic wounds can influence plasma miRNA levels and affect angiogenesis and cell migration. Later, some miRNAs were demonstrated to play critical roles in the healing process of DFU, one of the leading complications for DM patient death and disability. Yuan et al. [25] demonstrated that miR-203 upregulation impeded DFU wound healing in rats by repressing the process of epithelial to mesenchymal transition (EMT), and miR-203 knockdown counteracted this effect by promoting keratinocyte proliferation and migration and facilitating the EMT process through the reactivation of IL-8 (a target gene of miR-203) expression and the downstream IL-8/AKT pathway. Recently, Zhao et al. [26,27] demonstrated the essential function of miR-204-3p and miR-103 in wound margin tissues of DFU patients. Their results indicated that miR-204-3p and miR-103 act as positive and negative regulators in DFU wound healing, respectively, and further in vitro experiments demonstrated that miR-204-3p could promote the proliferation and migration while reducing the apoptosis of HaCaT cells by targeting Kruppel-like factor 6 (KLF6) gene expression [26], and miR-103 could inhibit the proliferation and migration while promoting the apoptosis of primary normal human dermal fibroblasts by targeting the regulator of calcineurin 1 (RCAN1) gene expression in a high-glucose environment [27]. These studies, along with our findings, enhance the understanding of miRNA roles in diabetic wound healing and may provide potential targets for clinical applications.

Our study highlights the role of miR-3138 and miR-3679-5p in keratinocyte migration under high-glucose/hypoxia conditions. Previous research has shown that miR-3138 upregulation promotes radioresistance in cervical cancer cells [28], while downregulation occurs in aortic tissues from aortic stenosis patients and in pediatric dysembryoplastic neuroepithelial tumors, where it may act as a tumor suppressor [29,30]. Interestingly, Chen et al. [31] reported that miR-3138 was upregulated in insulin-stimulated human umbilical vein endothelial cells (HUVECs) under a high-glucose environment and linked the deterioration of insulin resistance in the metabolic syndrome with miR-3138 upregulation and KSR2 (kinase suppressor of ras 2)/AMPK (AMP-activated protein kinase)/GLUT4 (glucose transporter isoform 4) signaling repression. This contrasts with the downregulation of miR-3138 in this study, suggesting the different expression patterns of miR-3138 in different tissues. On the other hand, earlier studies found that miR-3679-5p was significantly increased in patients with coronary artery calcification [32] and downregulated in saliva from pancreatic cancer patients [33], and thereby can serve as a potential biomarker for early disease detection. In addition, Li et al. [34] predicted that miR-3679-5p may bind to CYTOR (cytoskeleton regulator RNA) and collaborate with MACC1 (metastasis-associated in colon cancer-1) to constitute the CYTOR/miR-3679-5p/MACC1 axis in colorectal cancers, but the actual interaction between these molecules remained to be further elucidated. Furthermore, Wang et al. [35] recently reported that circulating exosomal miR-3679-5p can act as a novel biomarker for diagnosing chronic rhinosinusitis with nasal polyps (CRSwNP) and predicting postoperative recurrence in patients. Notably, miR-3679-3p is consistently upregulated in the current and previous studies. To the best of our knowledge, the roles of miR-3138 and miR-3679-5p in keratinocyte migration and diabetic wound healing have not been unraveled previously, and the present study reveals them for the first time. 

The predicted targets of miR-3138 and miR-3679-5p also provide insights into their roles in diabetic wound healing. PALM2-AKAP2 readthrough is a natural fusion protein functionally linked in a very unusual manner and implicated in plasma membrane dynamics, which may play a role in cell migration and wound repair [36]. SNX9, SNX18, and SNX30 constitute a separate subfamily of PX-BAR-containing sorting nexin (SNX) proteins. SNX30 is involved in endosomal sorting and may influence cellular trafficking and signaling pathways critical for wound healing [37]. ZNF365 is associated with DNA damage response and cell cycle regulation, which are vital for cell proliferation and migration [38]. On the other hand, DMXL1 is involved in vesicle trafficking and membrane fusion, processes crucial for cellular repair mechanisms [39]. PPP2R2A is a regulatory subunit of protein phosphatase 2, which plays a role in various cellular processes including cell growth and division [40]. TTC39C is implicated in lipid metabolism and may influence cellular energy balance and repair processes [41]. However, further verification using tools such as the luciferase reporter gene assay [26,27] should be helpful and is required. Together with the aforementioned KSR2 [31] and MACC1 [34], it is indicated that a single miRNA can regulate multiple various genes depending upon the tissue or environment in which it is expressed.

Other miRNAs, such as miR-132 [42], miR-129, and miR-335 [43], have been reported to promote keratinocyte migration during diabetic wound healing when overexpressed. miR-205 [44], miR-21 [18], miR-203 [45], and let-7b [46] are important regulators of keratinocytes during normal wound healing but appear less significant in diabetic wound healing. In addition, although miR-3138 and miR-3679-5p have not been further characterized in our model, their functions in diabetic wounds are still worth noting.

The study of miRNA expression patterns in specific cell types from clinical skin biopsies is challenging due to the diversity of cell types involved in wound healing, such as keratinocytes, fibroblasts, and macrophages. While cell models offer advantages, they also have limitations. Future in vivo studies are required to functionally characterize the roles of specific miRNAs in diabetic wound healing.

## 4. Materials and Methods

### 4.1. Cell Culture and Diabetic Wound Model

The line of HaCaT human keratinocyte cells, obtained from the Bioresource Collection and Research Center, Hsinchu City, Taiwan, was maintained in minimum essential media (MEM) supplemented with 10% fetal bovine serum (FBS), 100 U/mL penicillin, and 100 μg/mL streptomycin at 37 °C, 5% CO_2_, and 95% humidity. To simulate the hyperglycemic and hypoxic environment of diabetic wounds, cells were cultured in high-glucose (4.5 g/L, ~25 mM) Dulbecco’s Modified Eagle Medium (DMEM) (Catalog No. 12100-046, Gibco^®^, Thermo Fisher Scientific, Waltham, MA, USA) with 10% FBS under hypoxic conditions (95% N_2_, 5% CO_2_). Control cells were cultured in low-glucose (1 g/L, ~5.6 mM) DMEM (Catalog No. 31600-034, Gibco^®^) with 10% FBS under normoxic conditions (95% air, 5% CO_2_).

### 4.2. Scratch Wound Healing Assay

Scratch wound healing assays were performed as previously described [44,47]. Briefly, confluent HaCaT monolayers were scratched using a 10-μL pipette tip. After washing with cold medium, cells were incubated in fresh low- or high-glucose media under normoxic or hypoxic conditions. Wound closure was monitored daily and quantified as: [wound width (0 h) − wound width (x h)] / wound width (0 h) × 100%.

### 4.3. RNA Extraction and miRNA Microarray Analysis

Total RNA was extracted from wounded HaCaT cells at 24 h and 48 h post-wounding using TRIzol reagent (Life Technologies, Carlsbad, CA, USA) [48]. RNA quality and quantity were assessed using a NanoDrop 2000 spectrophotometer (Thermo Fischer Scientific, Waltham, MA, USA) and Agilent 2100 Bioanalyzer (Agilent Technologies, Santa Clara, CA, USA). Small RNAs (<200 nucleotides) were enriched using cutoff concentrators (Sartorius, Gottingen, Germany).

miRNAs were labeled with Cy5 dye (ULS^®^ microRNA Labeling Kit, Kreatech Diagnostics, Amsterdam, Netherlands) and hybridized to human miRNA OneArray^®^ 4.1 microarray chips (Phalanx Biotech Group, Hsinchu, Taiwan). Scanning and data normalization were performed using an Axon4000B scanner with GenePix^TM^ Pro software version 4.0 (Molecular Devices, San Jose, CA, USA). The fluorescence intensity for each spot was detected repeatedly, and background intensity with a GenePix^TM^ flag value < −50 was subtracted from the analysis. The intensities of probes were normalized by the 75% median scaling normalization method. The fold change (FC) of Cy5 intensities was calculated to determine the relative expression of miRNAs (high glucose and hypoxia vs. normal condition). Differentially expressed miRNAs were identified using criteria of |log2 FC| ≥ 1 and *p* < 0.01.

### 4.4. Quantitative Reverse Transcription PCR (qRT-PCR)

qRT-PCR was performed to validate microarray results [49]. cDNA was synthesized using the TaqMan^®^ MicroRNA Reverse Transcription Kit (Thermo Fisher Scientific). PCR was conducted using a MiniOpticon^TM^ real-time PCR system (BioRad, Hercules, CA, USA). The primers used for corresponding miRNAs are listed in Table 2. Relative miRNA expression was calculated using the comparative threshold cycle (CT) method, normalized to U6 snRNA.

### 4.5. miRNA Mimic and Inhibitor Transfection

miR-3138 mimic, miR-3679-5p inhibitor, and corresponding NC mimic and inhibitor were synthesized (RiboBio Co., Guangzhou, China) and were transfected into HaCaT cells (50 nM) using Lipofectamine 2000 (Invitrogen, Carlsbad, CA, USA) at a final concentration of 50 nM. Transfected cells were used for scratch wound assays 24 h post-transfection. Wound closure was assessed to evaluate transfection efficacy.

### 4.6. cDNA Microarray Analysis

For cDNA preparation and cDNA microarray analysis, cells transfected with miR-3138 mimic, NC mimic, miR-3679-5p inhibitor, and NC inhibitor were collected 48 h post-wounding. cDNA synthesis, labeling, and hybridization to human OneArray^®^ v6 microarray chips were performed according to manufacturer protocols. Chip scanning and data normalization were performed as described in miRNA microarray analysis [50]. The fold change of 3138M/NCM and 3679I/NT was calculated to determine possible genes affected by miR-3138 and miR-3679-5p. Differentially expressed genes were identified using criteria of |log2 FC| ≥ 1 and *p* < 0.05.

### 4.7. Bioinformatics Analysis

Differentially expressed genes were analyzed using Gene Set Enrichment Analysis (GSEA) for the KEGG pathway and GO term enrichment. A *p* value < 0.05 was set as the cutoff to select significantly enriched terms. Putative miRNA targets were predicted using TargetScan (http://www.targetscan.org/), miRDB (http://mirdb.org/miRDB/), and DIANA (http://diana.imis.athena-innovation.gr/DianaTools/) databases (accessed on 15 January 2024). Only targets consistently identified across all three databases were considered.

### 4.8. Statistical Analysis

Data are presented as mean ± standard deviation (SD) from at least three independent experiments. Statistical significance was determined using Student’s two-tailed *t*-test, with *p* < 0.05 considered significant.

## 5. Conclusions

This study serves as a foundation for understanding the roles of miRNAs in diabetic wounds. We have identified 22 miRNAs that differentially expressed at 24 h post-wounding and 27 miRNAs at 48 h post-wounding. Two key miRNAs, miR-3138 and miR-3679-5p, that influenced keratinocyte migration were particularly highlighted. Further research is needed to elucidate the exact regulatory mechanisms and develop effective and safe miRNA-based therapies for diabetic wound healing.

## Figures and Tables

**Table 1:** Differentially expressed miRNAs with significant variances under high-glucose and hypoxic environment.

miRNA	Normal	High Glucose & Hypoxia	High Glucose & Hypoxia/Normal (log_2_)	*p* Value	Time (h)
hsa-miR-4521 *	188	625	1.732	1.15 × 10^−4^	24
hsa-miR-1273g-3p *	914	2961	1.697	2.34 × 10^−3^	24
hsa-miR-3679-5p *^#^	461	1336	1.535	4.40 × 10^−5^	24
hsa-miR-4484 *	756	2007	1.409	1.28 × 10^−4^	24
hsa-miR-3676-3p	220	583	1.403	2.62 × 10^−4^	24
hsa-miR-4685-3p	481	1262	1.391	6.12 × 10^−4^	24
hsa-miR-4725-3p *^#^	325	818	1.329	6.00 × 10^−5^	24
hsa-miR-4656	634	1579	1.317	1.08 × 10^−4^	24
hsa-miR-204-3p	527	1273	1.272	1.34 × 10^−3^	24
hsa-miR-210	244	569	1.221	1.00 × 10^−4^	24
hsa-miR-4290	490	1140	1.219	6.30 × 10^−5^	24
hsa-miR-634	475	1094	1.205	7.30 × 10^−3^	24
hsa-miR-4763-5p	1522	3459	1.184	7.67 × 10^−4^	24
hsa-miR-766-3p	2283	4995	1.130	1.00 × 10^−6^	24
hsa-miR-4800-5p *^#^	356	767	1.108	1.18 × 10^−3^	24
hsa-miR-1976	556	1173	1.077	1.83 × 10^−3^	24
hsa-miR-1273a	347	710	1.031	1.30 × 10^−3^	24
hsa-miR-1225-3p	393	796	1.017	4.59 × 10^−4^	24
hsa-miR-5196-5p	744	1504	1.015	3.00 × 10^−6^	24
hsa-miR-205-3p *^#^	503	95	−2.409	3.00 × 10^−5^	24
hsa-miR-3138 *^#^	2110	968	−1.125	1.70 × 10^−5^	24
hsa-miR-4278 *^#^	298	140	−1.089	3.36 × 10^−4^	24
hsa-miR-4800-5p *^#^	1005	3319	1.723	5.00 × 10^−6^	48
hsa-miR-4725-3p *^#^	466	1180	1.341	4.00 × 10^−6^	48
hsa-miR-3679-5p *^#^	719	1576	1.133	1.30 × 10^−4^	48
hsa-miR-4454 *	8522	1442	−2.563	4.89 × 10^−4^	48
hsa-miR-205-3p *^#^	552	106	−2.386	3.40 × 10^−5^	48
hsa-miR-5191 *	1068	225	−2.245	2.00 × 10^−6^	48
hsa-miR-1260b	36,753	8477	−2.116	4.90 × 10^−5^	48
hsa-miR-4286	15,856	4075	−1.960	2.00 × 10^−6^	48
hsa-miR-5100	719	212	−1.759	5.28 × 10^−3^	48
hsa-miR-4284	23,429	7121	−1.718	9.06 × 10^−3^	48
hsa-miR-3138 *^#^	1336	480	−1.477	1.00 × 10^−6^	48
hsa-miR-4446-3p	890	321	−1.471	2.78 × 10^−4^	48
hsa-miR-3131	450	174	−1.368	4.80 × 10^−5^	48
hsa-miR-3676-5p	4522	1777	−1.347	3.32 × 10^−4^	48
hsa-miR-1587	7545	2973	−1.344	7.27 × 10^−3^	48
hsa-miR-933	611	243	−1.330	1.95 × 10^−3^	48
hsa-miR-4448	768	316	−1.283	1.30 × 10^−5^	48
hsa-miR-4278 *^#^	165	70	−1.240	2.40 × 10^−5^	48
hsa-miR-1280	33,934	14467	−1.230	1.60 × 10^−5^	48
hsa-miR-3182	218	95	−1.205	1.92 × 10^−4^	48
hsa-miR-4443	24,819	10835	−1.196	2.99 × 10^−4^	48
hsa-miR-718	309	144	−1.102	1.90 × 10^−5^	48
hsa-miR-4417	217	102	−1.094	8.10 × 10^−5^	48
hsa-miR-3196	1347	635	−1.085	9.52 × 10^−4^	48
hsa-miR-1260a	14,372	6902	−1.058	1.40 × 10^−5^	48
hsa-miR-1193	226	109	−1.048	6.00 × 10^−6^	48
hsa-miR-363-5p	2397	1160	−1.047	3.00 × 10^−3^	48

* miRNAs submitted to qRT-PCR verification. ^#^ miRNAs appeared repetitively at 24 h and 48 h post-wounding.

**Table 2:** Primers used in this study.

miRNA Target	Direction (F, Forward Primer; R, Reverse Primer)	Sequence (5’ to 3’)
hsa-miR-1273g-3p	F	attcaccactgcactccag
	R	gttggctctggtgcagggtccgaggtattcgcaccagagccaacctcagg
hsa-miR-205-3p	F	gcgatgatttcagtggagtg
	R	gttggctctggtgcagggtccgaggtattcgcaccagagccaacgaactt
hsa-miR-3138	F	atttgtggacagtgaggtaga
	R	gttggctctggtgcagggtccgaggtattcgcaccagagccaacactccc
hsa-miR-3679-5p	F	gtttgaggatatggcaggga
	R	gttggctctggtgcagggtccgaggtattcgcaccagagccaactcccct
hsa-miR-4278	F	ggctagggggtttg
	R	gttggctctggtgcagggtccgaggtattcgcaccagagccaaccaaggg
hsa-miR-4454	F	aaactggatccgagtcacg
	R	gttggctctggtgcagggtccgaggtattcgcaccagagccaactggtgc
hsa-miR-4484	F	ggcaaaaggcgggagaa
	R	gttggctctggtgcagggtccgaggtattcgcaccagagccaactggggc
hsa-miR-4521	F	attggctaaggaagtcctgt
	R	gttggctctggtgcagggtccgaggtattcgcaccagagccaacctgagc
hsa-miR-4800-5p	F	gcaagtggaccgaggaag
	R	gttggctctggtgcagggtccgaggtattcgcaccagagccaactccttc
hsa-miR-5191	F	gtccacaggataggaagaatga
	R	gttggctctggtgcagggtccgaggtattcgcaccagagccaacagcact
hsa-miR-634	F	ctaaaccagcaccccaact
	R	gttggctctggtgcagggtccgaggtattcgcaccagagccaacgtccaa
hsa-miR-766-3p	F	ggtggcttacacagctggaca
	R	gttggctctggtgcagggtccgaggtattcgcaccagagccaactgcctc
hsa-miR-933	F	agtgcgcagggagacc
	R	gttggctctggtgcagggtccgaggtattcgcaccagagccaacgggaga
hsa-miR-4725-3p	F	atggggaaggcgtcagt
	R	gttggctctggtgcagggtccgaggtattcgcaccagagccaaccccgac
U6	F	ttcctccgcaaggatgacacgc
	R	gttggctctggtgcagggtccgaggtattcgcaccagagccaacaaaaatat

## Data Availability

All data generated or analyzed during the current study are available from the corresponding author on reasonable request.
